# Supplementary material for: Neuroinvasion and Neurotropism by SARS-CoV-2 Variants in the K18-hACE2 Mouse
Source: Viruses. 2022 May 11;14(5):1020. doi: 10.3390/v14051020 (PMC9146514; doi:10.3390/v14051020)
Supplement: Supplementary file 1 [file viruses-14-01020-s001.zip › viruses-1685725-supplementary.pdf]

Supplemental Material

Supplemental Table S1. Antibodies and detection methods used for immunohistochemistry

Antigen	Antibody (clone)	Antigen retrieval	Antibody dilution, detection method
SARS-CoV NP	pAB rabbit[a]	CB pH 6.0, 98 °C, 20 min	1:6,000, ON, 4 °C EnVision+ (HRP, Rb)[f]
IAV	goat anti-IAV (H1N1; virions)[b]	TE pH 9.0, 96 °C, 30 min	1:200, 60 min, RT Rb anti-goat Ig/HRP[f]
Iba1	pAB rabbit anti-human Iba1[c]	CB pH 6.0, 98 °C, 20 min	1:750, 60 min, RT HRP EnVision+ (HRP, Rb)[f]
CD3	mAB rabbit anti-mouse CD3 (SP7)[d]	TE pH 9.0, 37 °C, 20 min	1:900, 60 min, 37 °C, OmniMap anti-Rb HRP[i]
CD45R	mAB rat anti-mouse CD45R (B220/RA3-6B2)[e]	CB pH 6.0, 98 °C, 20 min	1:800, 60 min, RT EnVision+ (HRP, Rat)[f]
GFAP	pAB rabbit anti-bovine GFAP[f]	CB pH 6.0, 98 °C, 20 min	1:600, 10 min, RT MACH4™ HRP[k]
APP	mAB mouse anti-Alzheimer precursor A4 (pre A4695) fusion protein (22C11)[g]	CB pH 6.0, 98 °C, 20 min	1:6,000, 60 min, RT MACH4™ HRP[k]
ACE2	mAB rabbit anti-human ACE2 (SN0754)[h]	CB pH 6.0 100 °C, 20 min	1:200, 10 min, RT EnVision+ (HRP, Rb)[f]
Cleaved caspase 3	mAB rabbit anti-cleaved caspase 3 (Asp175, 5A1E)[l]	TE pH 9.0, 98 °C, 20 min	1:200, ON, 4 °C EnVision+ (HRP, Rb)[f]

Legend: ACE2 - angiotensin-converting enzyme 2, APP - Alzheimer Precursor Protein A4, GFAP – glial fibrillary acidic protein, IAV – Influenza A virus, mAB – monoclonal antibody; pAB – polyclonal antibody; CB – citrate buffer; ON – overnight; RT – room temperature; Rb – rabbit; SARS-CoV NP – severe acute respiratory syndrome coronavirus nucleocapsid protein; TE – Tris-EDTA buffer;

Commercial providers:

[a]Rockland Immunochemicals Inc., Limerick, USA

[b]Meridian Life Sciences Inc., Memphis, USA

[c]WAKO, Osaka, Japan

[d]Spring Bioscience Corp., Ventana Medical Systems, Tucson, USA

[e]BD Pharmingen, Franklin Lakes, USA

[f]Agilent Dako, Glastrup, Denmark

[g]Merck Millipore

[h]Novus Biologicals, Centennial, USA

[i]Ventana Medical Systems Inc., Tucson, USA

[k]Biocare Medical

[1]Cell Signaling Technology

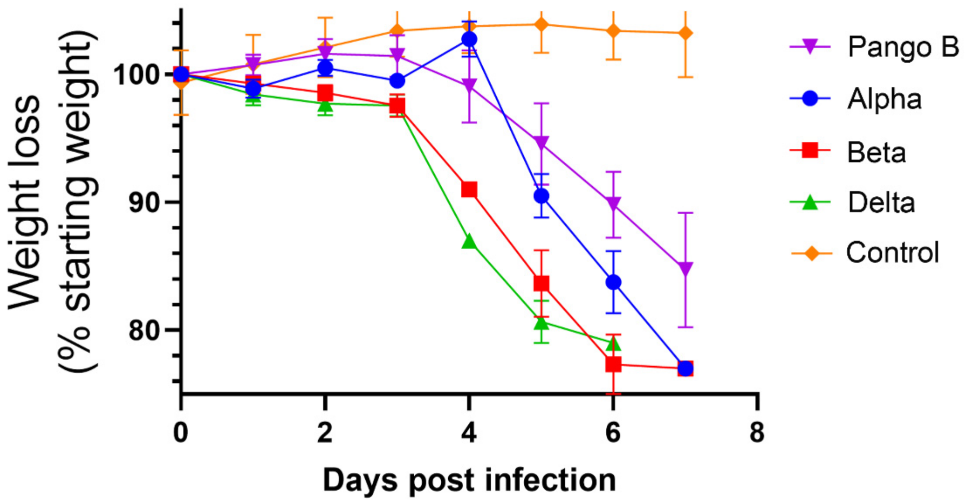

Figure S1. **Infection of K18-hACE2 mice with SARS-CoV-2 VOCs leads to substantial weight loss.** K18-hACE2 mice were infected intranasally with SARS-CoV-2 Alpha, Beta or Delta variant at 10^3 PFU (n=4 per group). Mice were monitored for weight loss at indicated time points and euthanized at days 5 and 6 post infection due to substantial weight loss (limit: 20% loss) or at the end of the experiment (day 7). The weights of Pango lineage B infected animals (10^4 PFU) from another experiment served for comparison. Data represent the mean value ± SEM.

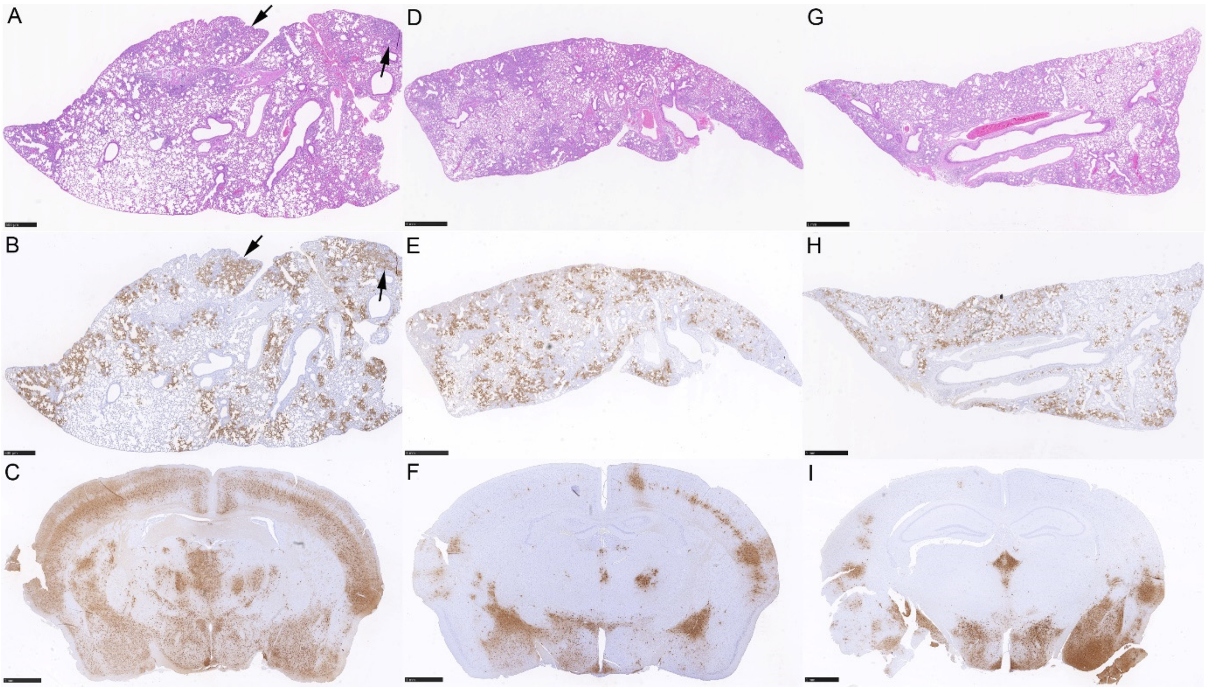

Figure S2. Infection of K18-hACE2 mice with SARS-CoV-2 VOCs leads to extensive pulmonary infection and spreads to the brain. K18-hACE2 mice were infected intranasally with SARS-CoV-2 Alpha, Beta or Delta variant at 10^3 PFU and euthanized at 5 or 6 days post infection (dpi) due to substantial weight loss (limit: 20% loss). Lungs and brains, overview. **A-C)** Animal infected with Alpha variant and euthanized at 6 dpi. **A, B)** Lung. **A)** Mild multifocal increase in interstitial cellularity (arrows) with activation of type II pneumocytes and occasional degenerate and sloughed off alveolar epithelial cells. **B)** There is extensive viral antigen expression in large patches of unaltered appearing alveoli and in more cell rich areas (arrows). **C)** Brain, coronal section at level of corpus callosum, caudoputamen, thalamus/hypothalamus and frontal cerebral cortex. There is extensive viral antigen expression in neurons in cerebral cortex, thalamic and hypothalamic nuclei as well as piriform area. **D-F)** Animal infected with Beta variant and euthanized at 6 dpi. **D, E)** Lung. **D)** Mild to moderate multifocal increase in interstitial cellularity with activated type II pneumocytes and degenerate and sloughed off alveolar epithelial cells. **E)** There is extensive disseminated viral antigen expression in large patches of unaltered appearing alveoli and in more cell rich areas. **F)** Brain, coronal section at level of corpus callosum, rostral hippocampus, caudoputamen, thalamus/hypothalamus and frontal cerebral cortex. There is multifocal patchy viral antigen expression in neurons in cerebral cortex, amygdala, thalamic/hypothalamic nuclei, piriform area. **G-I)** Animal infected with Delta variant and euthanized at 5 dpi. **G, H)** Lung. **G)** Mild multifocal increase in interstitial cellularity (arrows) with activation of type II pneumocytes and occasional degenerate and sloughed off alveolar epithelial cells. **H)** There is abundant disseminated viral antigen expression in variably sized patches of unaltered appearing alveoli and in more cell rich areas. **I)** Brain, coronal section at corpus callosum, hippocampus, caudoputamen, thalamus/hypothalamus and frontal cerebral cortex. There is multifocal patchy viral antigen expression in neurons in cerebral cortex, amygdala, thalamic/hypothalamic nuclei, piriform area. HE stain (A, D, G). Immunohistochemistry, hematoxylin counterstain (B, C, E, F, H, I).
